# Subjective Cognitive Decline and Related Cognitive Deficits

**DOI:** 10.3389/fneur.2020.00247

**Published:** 2020-05-19

**Authors:** Tong Si, Guoqiang Xing, Ying Han

**Affiliations:** ^1^Department of Neurology, Xuanwu Hospital, Capital Medical University, Beijing, China; ^2^The Affiliated Hospital and the Second Clinical Medical College of North Sichuan Medical University, Nanchong Central Hospital, Nanchong, China; ^3^National Clinical Research Center for Geriatric Disorders, Beijing, China; ^4^Center of Alzheimer's Disease, Beijing Institute for Brain Disorders, Beijing, China

**Keywords:** subjective cognitive decline, mild cognitive impairment, Alzheimer's disease, traumatic brain injury, diagnosis, intervention

## Abstract

Since late stage dementia, including Alzheimer's disease (AD), cannot be reversed by any available drugs, there is increasing research interest in the preclinical stage of AD, i.e., subjective cognitive decline (SCD). SCD is characterized by self-perceptive cognitive decline but is difficult to detect using objective tests. At SCD stage, the cognitive deficits can be more easily reversed compared to that of mild cognitive impairment (MCI) and AD only if accurate diagnosis of SCD and early intervention can be developed. In this paper, we review the recent progress of SCD research including current assessment tools, biomarkers, neuroimaging, intervention and expected prognosis, and the potential relevance to traumatic brain injury (TBI)-induced cognitive deficits.

## Background

In 1982, Reisberg (1) classified the course of Alzheimer's disease (AD) progression into the following seven stages according to the patients' clinical manifestations: (1) no cognitive decline, (2) very mild cognitive decline, (3) mild cognitive decline, (4) moderate cognitive decline, (5) moderately severe cognitive decline, (6) severe cognitive decline, and (7) very severe cognitive decline. The second stage was regarded as the earliest description of AD-related subjective cognitive decline (SCD). With the increased understanding of AD etiology detected by neuropathology, neuropsychology, pathophysiology, neuroimaging, and other techniques, the National Institute on Aging and Alzheimer's Association (NIA-AA) suggested that AD be subdivided into AD-preclinical stage, AD-MCI stage, and AD-dementia stage (2, 3). Of them, the preclinical phase of AD can be further divided into 3 substages: (1) amyloid deposition, (2) amyloid deposition and neuronal degeneration, and (3) neuronal degeneration and amyloid deposition with subtle cognitive decline (i.e., SCD stage). Until now SCD has had multiple names: subjective memory impairment (SMI), subjective cognitive complain (SCC), and subjective memory complaint (SMC) etc., with memory decline as the most prominent manifestation. With the recent research progress and understanding of the clinical symptoms of SCD from basic research and clinical studies, it is now recognized that visual space damage, language impairment, attention deficit, and other symptoms could co-exist in SCD patients. Therefore, “cognition” was the more appropriate word to describe the clinical characteristics of SCD rather than “memory” (4–6). Thus, the Subjective Cognitive Decline Initiative (SCD-I) Working Group has advocated the use of the name-SCD (6).

In 2014, the SCD-I working group proposed the SCD conceptual framework, and by redefining SCD, emphasized that the subjective perception of cognitive function decline of SCD patients is not a state and thus does not need to be confirmed by objective cognitive tests. Thus, the purpose of neuropsychological examination of SCD is to exclude the possibility of objective cognitive impairment. SCD could be characterized as memory impairment or multiple cognitive domains impairment. The SCD-I conceptual framework also described important clinical features and accompanying symptoms of SCD, emphasizing that key information—such as age of onset, onset manifestation, symptom fluctuation, accompanying symptoms, circumstantial evidence, and comorbidity—should be collected in future research. The conceptual framework has also emphasized that the following features can increase the likelihood of preclinical AD in individuals with SCD: subjective perceptive memory decline, onset of disease within 5 years, onset at over 60 years of age, worrying about cognition decline, or subjectively feeling worse memory than the same age group. Specificity of the SCD diagnosis increases if the following conditions are present: informative confirmation, carrying ApoEe4 allele, and with positive AD pathophysiological biomarkers. In short, SCD has gradually become better understood.

Stewart and others (4, 7) have suggested that toward the end of the preclinical stage of AD, the subjective perception of cognitive decline in the elderly is more sensitive than objective neuropsychological tests. Pathological changes in AD could have occurred years ahead of the manifestation of MCI (8), which suggests that there should be a “pre-MCI” phase before the manifestation of MCI. The cognitive functional deficits of the patients could be reversible to normal at this stage. Thus, SCD may be a critical stage for early diagnosis and intervention of AD.

SCD is an intermediate state between normal cognition and MCI that may predict the development of objective cognitive decline (9–11). AD is a progressive disease and an abnormal deposition of Aβ and Tau in the brain may occur 15–20 years before the clinical diagnosis of AD, whereas MCI often occur 2 to 3 years before the onset of AD (12). Therefore, the extended SCD concept fills the gap between the manifestation of initial decline in cognitive function of AD and occurrence of MCI. The inclusion of the SCD concept completes the full picture of AD development (from normal state to SCD to MCI and to AD) and provides the basis for the diagnosis and intervention of AD at the very early stage of the disease (2, 6, 13).

## Epidemiology

Epidemiological studies showed the prevalence of SCD ranged from 10 to 88%, with the highest prevalence found in the most advanced age group: 20% among people aged 65 years old and younger, 25 to 50% among people older than 65, and 88% among people older than 85 (4, 7, 14, 15). Jonker et al. (16) reported a 34.3% prevalence of SCD in community-based people aged 65–85 in the Netherlands, whereas Luck et al. (17) showed 12.3% prevalence of SCD in 117 of the 953 elderly participants (based on the general population sample). In a Spanish population-based cohort (ALFA project), 572 out of the 2670 participants studied reported SCD, showing a prevalence of 21.4% (18). Han et al. (19) explored the prevalence of SCD in the Shun Yi District in Beijing, China, by two standards. They found that the prevalence of SCD was 18.8% based on standard 1 (the Alzheimer's Disease Neuroimaging Initiative, ADNI) and 14.4% according to standard 2 (Jak/Bondi criterion), in their well-designed study. Another German study included 1,467 patients from clinics, wherein 792 (54.0%) reported SCD (20). In view of longitudinal studies, a multi-center longitudinal observational study (3-year follow-up) of the German Dementia Competence Network (DCN) revealed that 22% SCD patients progressed to MCI and 12% to AD, while only 3.7% subjects developed to a non-AD dementia (21). Some Swedish researchers found that in 122 SCD patients, over a follow-up period of 48 months, 39% declined cognitively and 10% converted to AD, in a clinical prospective single-center Gothenburg MCI study (22). Mitchell et al. (23) displayed that the conversion rate of SCD to MCI was 34.2% based on the community population over the mean period of 5 years through review of 28 studies. A recent 7-year follow-up study (24) showed that 212 subjects (109 patients with SCD) were considered: 26 out of 109 SCD subjects converted to MCI, 15 developed to AD, and 68 patients remained stable. A large-scale, 10-year-follow-up epidemiological survey conducted among 2,043 non dementia subjects showed that 372 old people developed dementia within 10 years (25, 26), of which 208 (55.9%) were diagnosed as AD. Cox regression analysis showed that SCD predicted all causes of dementia. From the results mentioned above, we can see that epidemiological datum of SCD differs from different studies because no common definition, criteria, and screening tools are available (27). Consequently, unified terminology and methodology and different populations (different age, community or clinic-based, etc.) are significant parts of further research.

## Clinical Characteristics

Subjective memory impairment (SMI), such as recent memory decline, is a common clinical manifestation of SCD. In comparison, only a few SCD patients reported subjective language impairment.

Emotional state and individual heterogeneities could affect SCD diagnosis. People with depression and anxiety tend to over worry about their cognitive ability, memory, or other cognitive decline (10, 25). These patients tend to regard normal physiological forgetfulness as severe memory decline and believe their cognitive functions are getting worse, increasing the physical and mental burden. And this kind of memory decline is often age-related, non-pathological, and without objective evidence. However, recent studies suggest that late-life depression is associated with increased risk of all causes of dementia, including AD (28, 29). Moreover, depression has been shown to be related to objective changes to brain structure and function (30), including gray matter abnormalities within frontal-subcortical and limbic networks (31) and loss of white matter integrity (32). Together, these findings suggest that arbitrary exclusion of persons with depression from SCD studies can result in incomplete understanding of the mechanisms by which SCD subjects progress to cognitive decline and dementia.

Furthermore, like depression and anxiety, enhanced stress levels and neuroticism may accelerate AD pathological progression and cognitive decline (33, 34). Therefore, it is often difficult to make the clinical diagnosis or differential diagnosis of SCD for people with neuro-emotional comorbidity. They are more prone to be considered for an exclusionary diagnosis, in which comprehensive factors including age, gender, education, experience, emotional state, etc. are considered. Including such patients in SCD research would require quantification of the impact of depression and other psychiatric symptoms so that they can be adjusted as potential moderator variables (33, 34).

As mentioned above, SCD is characterized by self-experienced cognitive decline. Thus, informant confirmation has been speculated to increase the accuracy of diagnosis for preclinical AD spectrum. Many researchers also reckon that self- and informant-reports represent complementary approaches (33).

## Neuropsychological Assessment

Mini-Mental State Examination (MMSE) and Montreal Cognitive Assessment (MoCA) are two widely used screening tools that evaluate overall cognitive function, with high sensitivity for detecting MCI and AD. However, for detection and assessment of SCD, their validations or alternatives are needed. Sahlgrenska Academy Self-Reposed Cognitive Impairment Questionnaire (SASCI-Q) has been used to distinguish SCD from normal cognitive population and to evaluate daily cognitive ability in several studies (37). The Memory Alteration Test (M@T) has been reported to have distinguished SCD from amnestic mild cognitive impairment (aMCI) and early AD with higher sensitivity and specificity in comparison with MMSE (37). However, M@T is not a comprehensive tool for evaluating the overall level of cognition (38, 39). The Subjective Cognitive Decline Questionnaire (SCD-Q) is a more informative test that can distinguish SCD patients from the normal cognition population (37, 40). The test places an emphasis on the concept of “cognitive-decline-as-chief-complaint in different sources.” The complementary confirmation of cognitive decline by both subjective and objective perception makes The Subjective Cognitive Decline Questionnaire (SCD-Q) a great screening tool in the diagnosis and prognosis of SCD (41). Other neuropsychological examination methods evaluated by different study groups include the Memory Complaint Questionnaire (MAC-Q) and Everyday Cognition (ECog) (42, 43). The most common cognitive impairment of SCD is episodic memory, followed by executive function. Researchers tried to screen SCD patients from elderly people with normal cognition using the Stroop Color Word Test (CWT) and found significant differences in the outcomes between the SCD and normal cognition individuals by using the CWT rather than general neuropsychological tests, which indicated that CWT could be used as an independent indicator of SCD in clinical management (44). Recently, Ismail et al. (45) developed the mild behavioral impairment checklist (MBI-C) questionnaire for SCD and MCI, with the aim to develop more accurate assessment tools for screening the clinical manifestations of patients at early-stage-AD. Some recent research indicated that neuropsychological assessment may aid clinical diagnosis, especially memory neuropsychological tests which may also be useful for evaluating the risk of progression to AD among SCD subjects (24, 46). However, most scales and evaluation methods are still in the development stage or in their initial testing stage. More large-scale, multi-center, and standardized studies are needed to determine the suitability for clinical application as well as unified screening tools.

## Biomarkers

Valid SCD biomarkers may reflect the intermediate stage between clinically normal older adults (yet still with potential SCD) and MCI individuals, which is in line with how SCD is currently conceptualized. Any established associations between SCD and biomarkers would further validate SCD as a valid stage prior to the onset of clinical impairment along the early AD trajectory (33). In this section, biomarkers of cerebrospinal fluid (CSF) are emphatically introduced.

Biomarkers of CSF are also significant auxiliary diagnoses and predictors of SCD. Biomarkers in the CSF of SCD patients may occur well before any significant changes in brain MRI. The low level of Aβ protein in CSF indicates increased amyloid protein deposition in the brain, while high levels of T-tau and P-tau proteins suggest neurodegeneration. Leoni et al. (47) reported that as the cognitive function declined, T-tau and P-tau proteins in CSF of SCD patients increased significantly whereas Aβ protein decreased significantly. These findings are consistent with the 2011-National Institute on Aging and Alzheimer's Association (NIA-AA) diagnosis standard for SCD (48). Although changes in Aβ protein and tau protein in the CSF of SCD population could be a possible diagnostic basis for SCD, other potential independent causative factors, such as severe anxiety and depression, should be excluded. A 5-year follow-up study of 149 patients (94 MCI patients, 55 SCD patients) conducted by Sierra-Rio et al. (49) showed that during the follow-up period, the ratio of CSFAβ42/P-tau decreased in 72.4% of the patients (MCI83%, SCD27%). Because all of these patients progressed to AD eventually, these results suggest that the decreased Aβ42/P-tau ratio in CSF could be a critical risk factor for SCD/MCI progressing to AD. Compared to tau protein or other related predictors, Aβ42 could better predict the progression of SCD to MCI or to AD as proposed by Van Harten et al. (50). They suggested that Aβ42 level is a better predictor for clinical progression of SCD whereas T-tau is a predictor for MCI progression. Another cohort study conducted in Holland by the same research group found that SCD patients with initial abnormal CSF levels of Aβ42, T-tau, and P-tau have increased the risk of progression into MCI or AD within 2 years by 16, 2.8, and 2.6 times, respectively, compared to those with normal levels, suggesting that low level Aβ42 is a strong predictive factor for cognitive decline in SCD patients (50). Indeed, patients with detectable biomarkers are more likely to undergo cognitive decline during the course of SCD progression.

Jia et al. (51) found that the exosomal concentrations of Ab42, T-tau, and P-T181-tau in AD patients were higher than those in aMCI and control groups and the level of each exosomal biomarker was highly correlated with that in CSF. Their multicenter study with two-stage design verified the consistence between CSF and blood exosomal biomarkers and confirmed that exosomal Ab42, T-tau, and P-T181-tau have the same capacity as those in CSF for the diagnosis of AD and aMCI. This kind of peripheral blood will play a significant role in non-invasive detection at the early stage of AD. However, these findings, including CSF and peripheral blood, still need further confirmation in longitudinal studies.

## Neuroimaging Examination

Many neuroimaging methods have been applied to diagnose SCD. Among them, Structural MRI (sMRI) can be applied to measure the volume and thickness of the patients' cortex (38). There is evidence to show that at SCD stage, the degree of the cortex atrophy is associated with the severity of cognitive impairment. Saykin et al. (52) reported that, compared to the normal population, the level of medial temporal lobes atrophy and frontal lobes atrophy of SCD patients is related to the degree of cognitive decline. Recent MRI studies also showed that people with MCI differed from very mild cognitive decline and normal controls in the right hippocampus volume (53, 54). People with low-volume right hippocampus and cognitive impairment are at greater risk of advancing to AD (53, 54). Overall, sMRI is expected to be a useful tool for diagnosis of SCD.

**Figure 1:**
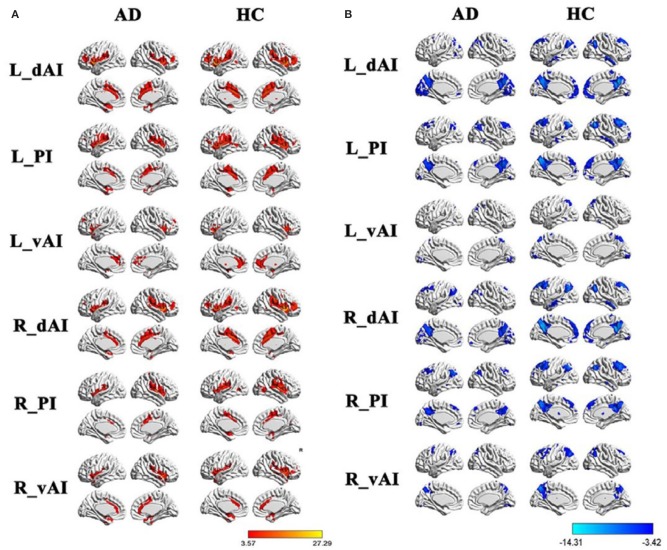
Within group resting-state functional connectivity (RSFC) analysis. **(A)** The positive RSFC patterns of the insular subregions in each group including healthy controls and AD patients. **(B)** The negative RSFC patterns of the insular subregions in each group including healthy controls and AD patients. PI, posterior insula; dAI, the dorsal anterior insula; vAI, the ventral anterior insula. Figure is reproduced from He et al. (35), with authors permission.

**Figure 2:**
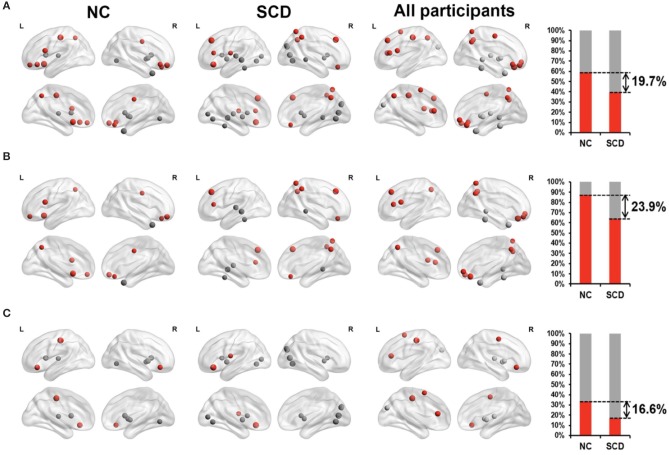
Relationship between degree of centrality (reflecting the dynamic functional connectivity) and neuropsychological tests in NC individuals (the first 2-columns), SCD patients (the second 2-columns), and all participants (the third 2-columns). The far-right column displayed the percentage of significantly correlated regions (*p* < 0.05) located in anterior (red) cortical and other (gray) cortical regions in the two groups. The nodes covered by red are located in anterior cortical regions while nodes covered by gray are located in posterior cortical regions or subcortical regions. **(A)** All the significant correlated regions (*p* < 0.05). **(B)** The regions in the DMN. **(C)** The regions out of the DMN. Figure is reproduced from Han et al. (36), with authors permission.

Functional MRI (fMRI) studies also show a weakened resting state in some of the brain regions of AD patients, such as the right hippocampus and insular subregions, indicating damage in these regions. Whereas, the increased activities in certain brain regions may suggest functional compensation in these areas at the early stage of AD (see Figure 1). Regarding SCD, the fMRI study by Ying's groups showed that (55), compared to the control group, there was a significant increase in the activity of the resting state in the following brain regions of SCD patients: inferior parietal lobule, right superior temporal gyrus, right inferior temporal gyrus, fusiform gyrus, and right posterior lobe of the cerebellum. This confirms the existence of compensatory mechanisms in spontaneous activities of SCD patients at the resting state. Another study suggested that patients with APOEε4 presented both increased and decreased functional connectivity as reflected in default mode network (DMN), which is correlated with clinical cognitive performances based on the regions of interest (ROI)-based functional connectivity analyses and voxel-based analyses (56). Thus, altered functional connectivity may be an early sign of cognitive decline. A resting-state fMRI study mainly revealed that SCD individuals had reduced correlations between centrality frequency (i.e., across the entire time window, the proportion of time that the hub appeared was defined as centrality frequency) of the anterior cortical regions and cognitive performance, compared with normal controls (NCs). In contrast, correlations between centrality frequency of the posterior cortical regions and cognitive performance were stronger in SCD participants than NCs. Besides, the alterations mainly concentrated on the anterior and posterior regions associated with the default mode network (DMN) (36). See Figure 2.

Glucose is an important energy source for brain function. Abnormal glucose uptake in different regions may be related to the extent of altered brain function. 18F-fluorodeoxyglucose position emission tomography (FDG-PET) can reflect changes in glucose metabolism in different parts of the brain (57). Scheef et al. (57) reported that, compared to the control group, metabolic rates in right precuneus and left parietal cortex are lower in SCD patients, while metabolic rates in medial temporal lobe and right para-hippocampal gyrus are higher, suggesting simultaneous abnormal energy metabolism and neuronal dysfunction in certain regions of SCD patients. And the decline in executive ability of SCD patients was associated with Aβ deposition in a Pittsburgh Compound B PET (PIB-PET) study (58). In recent years, breakthroughs in SCD research have been achieved by applying Flobetapir F-18 to Florbetapir-PET (amyloid-PET) techniques with improved sensitivity. Flobetapir F-18 has a higher affinity for Aβ accumulated in the brain and the results of Florbetapir-PET are highly consistent with that of Aβ in CSF that may be used as an early clinical diagnosis (59). Clark et al. (60), found an association between amyloid -PET imaging and the density of brain β amyloid protein. These findings show that PET can be an effective tool for detecting abnormalities in brains at SCD stage from function and Aβ deposit perspective.

Mallio et al. (61) demonstrated an epicentral disruption of structural connectivity in aMCI and AD around entorhinal and hippocampal regions, using diffusion-weighted imaging (DWI) consistent with the transneuronal spread hypothesis. Hereafter, researchers gradually paid attention to investigating brain network by Magnetic Resonance Diffusion Tensor Imaging (MR-DTI), and found that structural network properties might be preserved in patients with SCD but disrupted in aMCI stage, which may contribute to a better understanding of pathological mechanisms of AD (62). Li et al. (63) analyzed the DTI data in SCD patients by using Tract Based Spatial Statistics (TBSS), and found extensive white matter damage in SCD patients. These studies suggested that these pathological changes in SCD subjects were undetectable by conventional tests. Han et al. (64) also used DTI and graph theory approaches to demonstrate disrupted topologic efficiency of the brains' structural connectome of SCD. Thus, connectome-based biomarkers could be potentially used for detection of SCD in an elderly population. Another DTI study (65) revealed that rich club organization (some certain cortical regions highly connected to each other, with other regions referred to as peripheral) was destroyed, with less structural connectivity among rich club nodes in persons with MCI or AD but remained stable in SCD patients, which can show the development of AD and be viewed as a biomarker for early diagnosis. A recent systematic review including 16 studies (using neuroimaging tools containing Magnetoencephalography, structural and functional MRI, DTI, etc.) concluded that patients with SCD exhibited a significant abnormality of the brain network, compared to healthy controls, which was damaged in a similar approach as in Mild Cognitive Impairment (66). Magnetic Resonance Diffusion Kurtosis Imaging (DKI) is a relatively new technique that is an extension of DTI and is based on the non-Gauss-field of the water molecular diffusion. DKI can solve the problem of nerve-fibers-crossing and is more sensitive in observing subtle changes in brain white matter and gray matter (36, 56). Although only a few SCD studies reported using DKI, DKI will play an increasingly important role in the study of SCD in the future.

Together, comprehensive application of neuroimaging technology provides a valid way of capturing early brain alterations, and as such, these approaches may complement the absence of other neuropsychological tests and CSF biomarkers available for SCD diagnosis.

## Prognosis of SCD

The development of SCD could follow any of these three directions: (1) remission and symptoms fade away, (2) stabilized, (3) become worse and progress to MCI or AD. The conversion rate of SCD to MCI and AD in diagnosed SCD was reported to be 54.2%, of which 78.9% progressed to MCI in a 7-year follow-up study (4). In comparison, the risk of developing MCI or AD in the normal group was 14.9%, of which 71.4% developed to MCI. After adjusting for confounding factors, the risk for SCD group to develop to MCI or AD within 7 years was 4.5 times greater than that of no-SCD group. A meta-analysis by Mitchell et al. (23), showed that the risk of progression to AD in the elderly with SCD was 2 times greater than the elderly without SCD, with the annual conversion rate of SCD to MCI and AD as 6.6 and 2.3%, respectively. Importantly, many studies also suggested that low CSF Aβ42 and high t-tau or p-tau may predict cognitive decline (progression to MCI) in SCD subjects (67).

## Intervention

So far there is no FDA-approved pharmacologic interventions for SCD. A few supplements-based interventions have been reported. A 12-week dietary supplements treatment was conducted among 30 SCD patients (68), using active ingredients including Vinpocetine, uridine-5 monophosphate, hops, ginger, rosemary, Ashwaganda, grape seed, wild blueberry extract, L-alpha, Omega-glycerophosphate choline-3 phosphatidylserine, etc. The results showed significantly improved cognitive function in all subjects at the second week assessment, but no further improvement was found at the end of the study. Another report by Williams et al. (69) proposed that cognition training, vegetable intake, Mediterranean diet, Omega-3 fatty acid, physical activity, and non-physical leisure activities, when combined, could prevent cognitive decline. Among various nutritional markers related to aging and cognition, experts found that there may be an association between low-level Vitamin D and likelihood of functional deficits, such as coexisting or separate physical and cognitive decline in patients with subjective memory complaints (70). Researchers also found that a 4-week simultaneous memory training and aerobic exercise program can contribute to improving memory, reasoning abilities, and attention in a recent study (71). At the 2017 London Alzheimer's Association International Conference (AAIC), “Lancet” International Disease Prevention, Intervention and Nursing Committee released a new analysis system that identified nine modifiable risk factors, including <12 years of education, hypertension, obesity, hearing loss, depression, diabetes, lack of exercise, smoking, and social isolation. Most of these risk factors could be modified from childhood to middle age and account for 35% of AD. Using the stratified cluster random sampling method, Han et al. (19) investigated the prevalence and risk factors of SCD in Shun-Yi District of Beijing, China, and found high prevalence of SCD among people who were of an old age, had a low education, less social support, and daily drinking habits, highlighting these as independent risk factors. Because most of these factors are modifiable except age, the preservation and mobilization of brain plasticity by cognitive training, healthy diet, active aerobic exercise, smoking cessation, alcohol restriction, control of diseases such as diabetes, hypertension, dyslipidemia, anxiety and depression, enriched social activities, alone or combined, could be potential directions of intervention. Recently, numerous systematic reviews and meta-analyses (72) indicate that non-pharmacologic intervention (NPI) can be an effective intervention for SCD, particularly, cognitive interventions can benefit objective cognitive functioning, offset some normal age-related cognitive decline, support productive aging, and enhance quality of life for individuals who may otherwise develop MCI or dementia, such as mind-body exercise, especially Tai Chi, a well-known type of traditional Chinese martial arts (73). Some researchers also found that ketogenic diet may be another early non-pharmacologic intervention in AD (74). NPI could be more useful than medication due to its cost-effectiveness, lack of side effects, the fact that it can be readily adapted by a wide diversity of appropriately trained and experienced health professionals (72), and it is administrable before symptom onset (75). However, there is still a lack of high-quality research (such as randomization, blinding of participants and clinicians, use of “intention to treat” analysis for incomplete outcome data, etc.) in this direction (76).

## Relevance to Traumatic Brain Injury-Induced Cognitive Deficits

The past decades have seen traumatic brain injury (TBI) rush into the forefront of neurology due to the increasing incidence of falls, motor vehicle accidents, sports injuries, and wars (77). TBI is defined as altered brain function or other evidence of brain pathology caused by an external force (jolt to the head, blow, or other such penetrating head injury) and, more importantly, has been increasingly recognized as a risk factor for cognitive decline, dementia, and AD (78). Numerous studies also showed that there was a linear relationship between TBI severity and cognitive consequences after TBI, more specifically, the greater the severity, the greater the outcomes. To date, some links and details of mechanisms by which TBI leads to cognitive impairment remains to be fully elucidated. However, it is believed that excitotoxicity mediated by an overproduction of excitatory neurotransmitters like glutamate extracellularly, is hypothesized to be the sentinel event after TBI (79). Neuroinflammation is also activated in response to TBI, and has both beneficial and hazardous consequences. Some of the released pro-inflammatory cytokines, anti-inflammatory cytokines, and chemokines may induce Aβ plaque deposition in the brain (80, 81). After TBI, some brain areas have been shown to suffer from hypoxic damage and secondary ischemia (82), which is involved in the pathogenesis of AD by accelerating the accumulation of Aβ and increasing the hyper-phosphorylation of tau, resulting in chronic neurodegeneration (82). Furthermore, a growing number of studies have focused on the cerebrovascular link between TBI and AD. Cerebrovascular outcomes of TBI include edema, hemorrhages, vasospasms, changes in cerebral blood flow (CBF), blood-brain barrier (BBB) breakdown, coagulopathy, and chronic inflammation (83). TBI is a trigger of neurodegeneration and is a useful model for studying certain pathological features of AD, such as Aβand tau deposition. Conversely, Aβ and tau release can lead to cerebrovascular injury, and their accumulation around cerebral micro-vessels has a deleterious chronic impact (84, 85). In addition, pericyte dysfunction and alterations in endothelial cell after TBI are contributors to the neuropathology and cognitive deficits. Indeed, TBI is intimately related to cognitive decline.

As mentioned above, patients who report a history of TBI are more likely to precede an onset of AD and enough data support some overlapping distributions of pathology between them. In each of these two disorders, the final common pathway into clinical symptomatology includes the malfunctioning and death of neurons. Therefore, it is considered that, for AD investigators, it might be better to pay attention to different research directions that potentially offer opportunities to outflank TBI rather than frontally assault, AD, because of failure of related clinical trials before, in more depth, TBI as a possible model could contribute to better understanding the puzzle of AD. Specifically, a TBI provides investigators with the opportunity to induce many of the shared neuropathologies by animal models for TBIs or study them in patients who have suffered a head injury. TBI also offers basic and clinical investigators a temporally condensed microenvironment potentially reflecting within days and weeks of neuropathological progressions that can be studied in AD animal models or patients only by cross-sectional sampling of at risk subjects chosen from a population spread across decades (78). And we speculate that some neuropathologies may be observed at early stages of cognitive impairment because head injuries can be viewed as a starting point. Also, we are supposed to focus on prevention at the early stage, SCD, due to disappointing outcomes of AD therapy (78, 86, 87). Thus, TBI may be a definite breakthrough-point for studies of SCD. Additionally, it is noteworthy that subjective cognitive decline complaints after TBI and post-traumatic stress disorder (PTSD) have frequently been reported (88, 89), however, high anxiety and low mood resulting from TBI and PTSD may significantly influence subjective cognitive function, especially subjective appraisal of memory without any objective decline. Thus, clinicians should take that into account when distinguishing between TBI-induced affective disorders (anxiety, depression, etc.) and AD-related SCD, especially in old patients (89). Overall, there are differences and interrelations between TBI-induced SCD and AD-related SCD, and TBI is a significant focus of dementia research, with both basic and clinical research.

A summary of recent studies of TBI and cognitive decline is listed in Table 1.

**Table 1:** Summary of recent TBI studies with cognitive decline.

**References, Country**	**Design**	**(1) Injury severity** **(2) Diagnosis method**	**Assessment times (post injury)**	**Assessment tool**	**Conclusion**
Chen et al. (90) China	Prospective phase II pilot study	(1) Mild TBI (2) not reported	Baseline: 1 day t1: 7 ± 2 days t2: 28 ± 4 days t3: 84 ± 10 days	MMSE, Cognitive Abilities Screening Instrument	Cerebrolysin improved the cognitive function of patients with mild TBI at the third month after injury
Covassin et al. (91) USA	Prospective cohort	(1) sport-related concussion (2) American Academy of Neurology graded concussion assessment;	Baseline: preseason t1: 2 days t2: 7 days t3: 14 days	Post-Concussion Assessment and Cognitive Testing	The outcomes supported sex differences in memory and symptoms, age differences in memory and an interaction between sex and age on postural stability after concussion
Dikmen et al. (92) USA	Prospective longitudinal	(1) Mild TBI (2) GCS, CT	Baseline: 1 month t1: 12 months	Wechsler Adult Intelligence Scale, Halstead-Reitan neuropsychological battery, Simple Reaction Time Test, Finger-Tapping Test	Although the majority of neuropsychological and functional differences abated by 1 year, reporting three or more post-traumatic symptoms remained for around 50% of individuals
Failla et al. (93) USA	Prospective cohort	(1) Severe (2) GCS, GOS	Baseline: 6 months t1: 12 months	Functional Independence Measure-Cognition, Trail Making Test A and B, Digit Span test, Rey-Osterreith Complex Figure Test, II California Verbal Learning Test (Edition 2), Delis-Kaplan Executive Function System, Stroop, Controlled Oral Word Assoc Test	The results revealed that genetic variation within DRD2 was associated with cognition recovery post TBI
Farbota et al. (94) USA	Prospective cohort	(1) Moderate to severe (2) GCS, MRI	Baseline: 2 months t1: 12 months t2: 4 years	Controlled Oral Word Assoc Test, Wide Range Achievement Test, Reading Subtest (Edition 3), Wechsler Adult Intelligence Scale (Edition 3), Trail Making Test A and B	The data showing progressive white matter damage for several years after TBI supported the hypothesis that TBI should be regarded not as an isolated incident but as a prolonged disease state
Kontos et al. (95) USA	Prospective longitudinal	(1) Mild TBI (2) GCS	Baseline: pre-injury t1: 1–7 days t2: 8–20 days	Military Immediate Post-Concussion Assessment and Cognitive Testing	Decreases in neurocognitive performance and increased mild TBI symptoms were observed in the first 1 day to 7 days after combat-related mild TBI, and a history of blast-related mild TBI may influence these effects
Meier et al. (96) USA	Prospective longitudinal	(1) sport-related concussion (2) MRI	Baseline: 1.41 ±0.94 days t1: 8.73 ± 2.19 days t2: 31.46 ± 4.67 days	Automated Neuropsychological Assessment Metrics	The outcomes showed the evidence of reduced cerebral blood flow in human concussion and subsequent recovery, which may predict consequences following concussion
Ponsford et al. (97) Australia	Prospective longitudinal	(1) Mild TBI (2) GCS	Baseline: 0–48 h t1: 1 week t2: 3 months	Immediate Post-Concussion Assessment and Cognitive Testing	Patients with mild TBI were more likely to report ongoing memory and concentration problems in daily activities after trauma recovery
Register-Mihalik et al. (98) USA	Prospective longitudinal	(1) sport-related concussion (2) GCS	Baseline: preseason t1: 2.36 ± 1.41 days	Automated Neuropsychological Assessment Metrics	The data suggested that the multifaceted battery is more sensitive than any single measure in clinical concussion measures, and sensitivity to balance and neurocognitive impairments was low for each individual measure
Robertson and Schmitter-Edgecombe (99) USA	Prospective longitudinal	(1) Moderate-severe (2) GCS, post-traumatic amnesia	Baseline: 45.00 ± 35.14 days t1: 280.11 ± 104.11 days	Symbol Digit Modalities Test, Trail Making Test, Rey Auditory Verbal Learning Task, Wechsler Adult Intelligence Scale, Controlled Oral Word Assoc Test	Outcomes showed that the error-monitoring performance of patients with TBI was significantly poorer than controls at both baseline and follow-up
Sandhaug et al. (100) Norway	Prospective cohort	(1) Moderate, severe (2) GCS, loss of consciousness	Baseline: 3 months t1: 12 months t2: 25 months	Functional Independence Measure-Cognition, Glasgow Outcome Scale Extended	Functional Independence Measure and Glasgow Outcome Scale Extended in TBI research were more relevant for assessment of the functional recovery in a sub-acute phase than in later stages of TBI recovery
Schmitter-Edgecombe et al. (101) USA	Prospective longitudinal	(1) Moderate, severe (2) GCS, post-traumatic amnesia	Baseline: 41.85 ± 25.68 days t1: 289.00 ± 85 days	Rey Auditory Verbal Learning Task, Controlled Oral Word Assoc Test, Wechsler Adult Intelligence Scale, Letter Number Sequencing Test, 5-point test, self-ordered pointing test, Trail Making Test	TBI patients showed recovery in both content and temporal order memory for activities during the first year, however, activity memory performances remained poorer than controls at follow-up. Greater self- and informant report of everyday memory difficulties was relevant to poorer temporal order memory
Sours et al. (102) USA	Prospective longitudinal	(1) Mild TBI (2) GCS, CT	Baseline: 7.7 ± 2.4 days t1: 36.0 ± 8.2 days	Automated Neuropsychological Assessment Metrics	The results exhibited that reduced interhemispheric functional connectivity may result in the subtle cognitive deficits in mild TBI patients with serious symptoms
Vanderploeg et al. (103) USA	Prospective longitudinal	(1) Moderate to severe (2) post-traumatic amnesia, loss of consciousness	Baseline: 32.4 ± 12.8 days t1: 6 months t2: 12 months	California Verbal Learning Test	The results were in support of an impaired consolidation hypothesis as the primary deficit underlying memory impairment in TBI
Veeramuthu et al. (104) Malaysia	Prospective longitudinal	(1) Mild TBI (2) post-traumatic amnesia, loss of consciousness, CT, GCS	Baseline: 4.4 ± 8.3 h after gain of consciousness t1: 6 months	Neuropsychological Assessment Battery-Screening Module	The uncomplicated mild TBI group exhibited slower recovery, especially in tasks of memory, visuospatial processing, and executive functions, at follow-up, compared with patients with complicated mild TBI
Wang et al. (105) China	Prospective longitudinal	(1) Moderate to severe (2) GCS, CT, MRI	Baseline: 5.86 ± 4.54 years t1: 6 months following baseline	Functional Independence Measure-Cognition	The results demonstrated that the umbilical cord mesenchymal stem cell transplantation improved the neurological function and self-care in patients with TBI
Wylie et al. (106) USA	Prospective cohort	(1) Mild TBI (2) GCS, CT, loss of consciousness	Baseline: 2.0 ±0.9 days t1: 8.7 ± 1.2 days	Immediate Post-Concussion Assessment and Cognitive Testing	The data provided neuroimaging evidence for working memory deficits during the first week following mild TBI. Patients with persistent cognitive symptoms after mild TBI had increased requirement for posterior cingulate activation to finish memory tasks at 1 week following a head trauma
Zaninotto et al. (107) Brazil	Prospective longitudinal	(1) Moderate to severe (2) GCS, MRI	Baseline: 6 months t1: 12 months	Rey-Osterreith Complex Figure Test, Wechsler Adult Intelligence Scale (Edition 3), Grooved Pegboard Ask	The use of citicoline for 3 months, compared with placebo, did not result in enhancement in functional and cognitive status

## Conclusion

The concept of SCD could shift the ineffective late-stage diagnosis and ineffective treatment of AD into a more effective prophylactic strategy. To better explore the field of preclinical AD, several significant issues should be resolved. First, standard terminology and assessment practices should be unified and adopted. Second, due to SCD's heterogeneity and subjectivity, comprehensive assessment methods can enhance the predictive validity of SCD as a marker for preclinical AD, including neuroimaging, CSF, blood, informants' complaints, and so on. Third, intervention trail, especially well-designed NPI for patients with SCD, is still a hotspot. Last but not least, studies of TBI animal models or patients may contribute to better understanding of more complex mechanisms of SCD, resulting from many neuropathologies similar to AD. Consequently, large-cohort, multi-centered, and longitudinal studies are needed to achieve more breakthroughs in the field of cognitive impairment.

A summary of the main findings or conclusions for each section is listed in Table 2.

**Table 2:** Summary of main findings or conclusion for each section.

**SCD**	**Main findings or conclusion**
Concept proposing	AD-related SCD is an intermediate state between normal cognition and MCI that may predict the development of objective cognitive decline
Epidemiology	Epidemiological datum of SCD differ from different studies because of lack of unified terminology, criteria, screening tools and so on
Clinical Characteristics	Memory decline is a common clinical manifestation (excluding depression, anxiety and so on)
Neuropsychological Assessment	Most neuropsychological scales are still only for research
Fluid biomarkers	CSF and peripheral blood are meaningful predictive biomarkers for SCD
Neuroimaging Examination	Comprehensive application of neuroimaging technology (PET, fMRI, DTI, etc.) contribute to the diagnosis of SCD and better understanding the pathology
Prognosis of SCD	Remission, stabilized and progressing to MCI or AD
Intervention	NPI can be effective intervention for SCD
Relevance to Traumatic Brain Injury-induced cognitive deficits	1. Although TBI is a potential risk factor of AD and affective disorders, currently, it is not clear how TBI-related cognitive deficits are associated with SCD and AD. 2. SCD is often observed in patients with a history of TBI. 3. People with TBI and AD also share similar pathological alterations. 4. If a relationship between TBI (-related SCD) and dementia can be established, better diagnosis and early prevention strategy could be designed to prevent TBI-related SCD and dementia

## Author Contributions

TS wrote the first draft of this manuscript. YH and GX critically revised the manuscript. All authors read and approved the final manuscript.

## Conflict of Interest

The authors declare that the research was conducted in the absence of any commercial or financial relationships that could be construed as a potential conflict of interest.
